# “Interstellar Wanderers:” Digital Support for Teaching Place Value Within the Activity Approach Framework

**DOI:** 10.11621/pir.2023.0402

**Published:** 2023-12-01

**Authors:** Elena Vysotskaya, Anastasia Lobanova

**Affiliations:** a Psychological Institute of Russian Academy of Education, Moscow, Russia

**Keywords:** concept acquisition, orientation procedures, computer support, positional counting, multi-digit number, place value.

## Abstract

**Background:**

The majority of mistakes students make while using “well-learned” decimal counting, can be attributed to their miscomprehension of the structure and arrangement of the “positional” numeral system. Digital support may merely serve an illustrative and training function, or it may provide the special environment for locating the problem of positional counting as a part of meaningful actions by the students. Following the Activity approach, we aimed to scaffold the students’ own learning actions, in such a way as to reveal the origin of the multi-digit number concept. Thus, we used counting in other-base systems as a way for students to reconsider the reasoning behind familiar operations in the most common base-ten system.

**Objective:**

The purpose of this paper is to present the approach to computer support which we have designed, based on our analysis of the activity content related to the multi-digit number concept, and to discuss some preliminary results of the first training series.

**Design:**

The approach to educational environment design developed within the Learning Activity theory defines the ways in which a computer becomes essential. The computer should provide a transparent interface which allows students to perform transformations with objects which will react accordingly. The additional opportunities to perform concept-mediated orientation procedures should also be scaffolded by digital means. For the purposes of our study, the computer-based educational module “Interstellar wanderers” was designed. Four groups of students from 2nd to 5th grade (8-12 years of age, 20 children in total) participated in the experimental computer-based lessons (over 30 hours); classroom observations, videotaped discussions, and logs of students’ individual work in the computer simulation were used for analysis.

**Results:**

The preliminary results of the experimental teaching showed that the computer support which we developed may scaffold students’ progress toward acquisition of the multi-digit number concept through a reflective re-thinking of the well-learned decimal system. Yet further research is needed to get a quantitative analysis of students’ performance.

**Conclusion:**

The general principles of computer support design based on the Activity approach in education (Galperin, Davydov, & Talyzina) demand a thorough analysis of the origin of the concept being studied, as well as the design of appropriate content and means of students’ actions and corresponding contexts and tasks. The digital means which we designed to support students’ learning activity, are in demand and bring promising results.

## Introduction

The place value concept is fundamental for mastering mathematics: it stands for the rationality of arithmetic operations with multi-digit numbers (Moeller et al., 2011). Even those primary school students who can already count up to 100 experience difficulties with the digit structure of numbers (Fuson, 1990; Kamii & Joseph, 1988; Thompson, 2000). “Positional” counting in the primary school years is often taught with a memorable “rhyme,” used for one-by-one naming of some counted units, and accompanied by the children’s rhythmical movements (hopping, going up and down the stairs, etc.). The children do not comprehend the part-whole relations of the “denominals” behind naming the numerals: the “2” in “25” is not perceived as 20, although the counting materials can be enumerated correctly (Ross, 1985).

Researchers have distinguished various stages of children’s comprehension of place value as they can be assessed throughout the primary school grades, and analyzed the difficulties which students experience on the way (Fuson, 1990; Lengnink & Schlimm, 2010; Ross, 1985). Understanding the place value concept is known to be predictive of the overall mastery of mathematics (Desoete, 2015), and thus, the development of feasible ways to support students’ comprehension is an urgent problem. There are researchers who focus on transcoding issues between the symbols and naming of multi-digit numbers in different languages (Hewitt & Alajmi, 2023) and on “number sense” in general (Siegler, 2016; Tikhomirova & Malykh, 2021). The central feature of their instruction theories, however, is the necessity of “bundling” (Lengnink & Schlimm, 2010; MacDonald, 1972; Mix et al., 2019), which directly reflects the composition of the multi-digit number. The preliminary introduction of the place value concept is mostly limited to two-digit numbers: the relationship between “ones” and “tens” are examined in detail, including the special operations of “trades” or “carries” needed for subtraction and addition, and then transferred to other digits (hundreds, thousands, etc.).

Different visual aids to “boost” students’ understanding the place value concept are used widely, and their character and effectiveness are considered in many studies (Fuson & Briars, 1990; Herzog et al., 2019; Lafay et al., 2023; Mix et al., 2019). The most popular content among the various computer support designs for teaching about place value and operations with multi-digit numbers, is vivid illustration of separate objects grouped by tens (hundreds, thousands) in the decimal system^1^ (Richey et al., 2021). However, the role of “visual aids” for developing students’ comprehension of place value remains questionable. The bundles of objects grouped by tens appear ready-made, and the operation of bundling is demonstrated and copied by junior “students,” but the conceptual origin of positional counting remains hidden. It is thus a reasonable conclusion that children cannot simply “swallow” these “ready-made” concepts, which look efficient to adults; they need to reconstruct them on their own (Kamii & Joseph, 1988; Lai & Fung, 2018).

Sometimes the appeal to system counting is prompted by comparing it with wellknown non-system measurements (time, weights, calendar, money), as they were established ages ago. Another feasible approach is to reconsider the basis of familiar numerals through other-base systems. An obvious way is to use other-base systems to support students’ generalization of the place value principle, which has already been learned through decimal counting. Students are supposed to transfer the concept of place value to another-base system, either as an additional range of tasks for 5th graders (Gelfman & Kholodnaya, 2019; Gelfman et al., 1991; MacDonald, 1972; Vilenkin et al., 2023), or as an introduction to computer science and IT learning in high school. This approach to the development of place value comprehension is sometimes considered applicable not only for school students, but even for “grownup” prospective teachers (Thanheiser & Melhuish, 2019).

An alternative method is to introduce other-base systems before examining the decimal system’s structure. As examples of the developmental instruction curricula have shown, in primary school it is possible to pose the problem of invention of positional numeration through working with measuring tools of different sizes (Aleksandrova, 2009; Davydov et al., 1994; Zaharova & Feshenko, 1992). The students’ task is to measure and build up magnitudes with a special set of measuring tools, which allows precise operations with large and small values at the same time. The ratio between “neighboring” measurements may either change, or be invariant. The latter serves to build up the multi-digit number in the system of counting with a corresponding base. This “twist” has already proved to be fruitful, and appropriate educational materials have been designed. Students in these classes mastered actions (measuring, comparison, simple calculations with carries, etc.) in different numeral systems and performed fluent transition to decimal counting as a particular case of positional notation.

This approach, from our point of view, is productive for analyzing the development of the multi-digit number concept; it allows students to perceive the evolution of methods of counting and to reconstruct the origin of the numeric systems. The construction of the multi-digit number for other-base systems may also provide the content for digital support of the initial steps in learning mathematics, as well as for the transition period between primary and secondary education.

Our general aim was to examine the content and means of actions substantially linked to the formation of the proper multi-digit number concept, which are to be scaffolded by the computer. We strove to set the problem of multi-digit number arrangement within a meaningful context, and to provide our educational design approach with computer support. The appeal to the structure of the multi-digit number, which reflects the way it was obtained, is assumed to be the actual means of solving various tasks in the corresponding domain — and thus, a sequence of evolving learning situations may be based on it.

According to Davydov (2008), the origin of a concept may be revealed through students’ own actions within a learning task by immersing the students in a problem-solving context, where the multi-digit number has not been introduced ready-made, and the method for reaching the solution is not demonstrated by the teacher through simple visual aids. The choice and design of the context and the material to scaffold the students’ own actions which can reveal the meaning of the number notation, are needed.

The main idea of an educational module using other-base counting for students familiar with decimal counting, is to provoke “active” reflection on the foundation for the numeral systems. The necessity to count and calculate in other-base systems sets up a contradiction between the “conceptual” way of solving problems and “natural” familiar decimal numeration, and maintains the relevance of orientation procedures. According to Galperin (1992; 2007, see translated Galperin’s lectures in Engeness, 2021) and Talyzina (2018), the design of tasks which require overcoming salient features of the situation, is the indispensable condition for “conceptual” thinking development. Therefore, the priority task for an educational designer who follows the Activity research tradition, would be the construction of learning situations and the choice of relevant student actions for the corresponding concepts formation.

Thus, the goal of the current study was to develop and test a sample educational module, including the digital environment, aimed at students’ acquisition of the place value concept in general, and at conscious operations with decimal numbers in particular.

## Methods

For the purposes of our study, we designed a computer-based educational module called “Interstellar wanderers.” The model builds upon students’ reflections on the basics and principles behind familiar decimal counting, which is provoked by the contradiction between calculations in other-base systems. The experimental teaching was conducted in four small groups (a total of 20 students, from 8 to 12 years old). The lessons (of 40-50 minutes) were held twice a week as an elective module (over 30 lessons for each group); thus, students’ participation was voluntary. The digital support ran on the web within a standard browser and was developed using HTML/ JavaScript. The materials were deployed on the web-based learning management system (https://counter.digitar.ru), which logs all the participant’s actions.

For each lesson, students received a small homework assignment: several “practical” tasks, set within the same digital environment, which required them to move forward through “training flights,” based on their progress along the outlined trajectory of the concept’s development. The problem-solving could not be reduced to unconscious reproduction of the classwork tasks; the development of each solution demanded that they externalize the orientation procedures anew. These solutions, as well as the mistakes made, were then discussed in the class. Classroom observations, students’ materials, tests, video-taped classroom discussions, and the logs of students’ individual homework in the digital support software were used as data sources. The authors of the educational module observed the lessons (through videos, online, or in person) to ascertain that the students’ activity was relevant to the intended one.

Students’ promotion within the module was scaffolded by the digital environment with challenging tasks, educational texts, and the special externalized modeling space. The software thus supported the “executive” actions, and the “orientation” part was performed by the students in materialized form (Galperin, 2007; Engeness, 2021), which called for laying counting tokens on the table (an analogue of the real “counting table” was provided in the digital environment to support students’ joint inquiry online).

**Figure 1.**
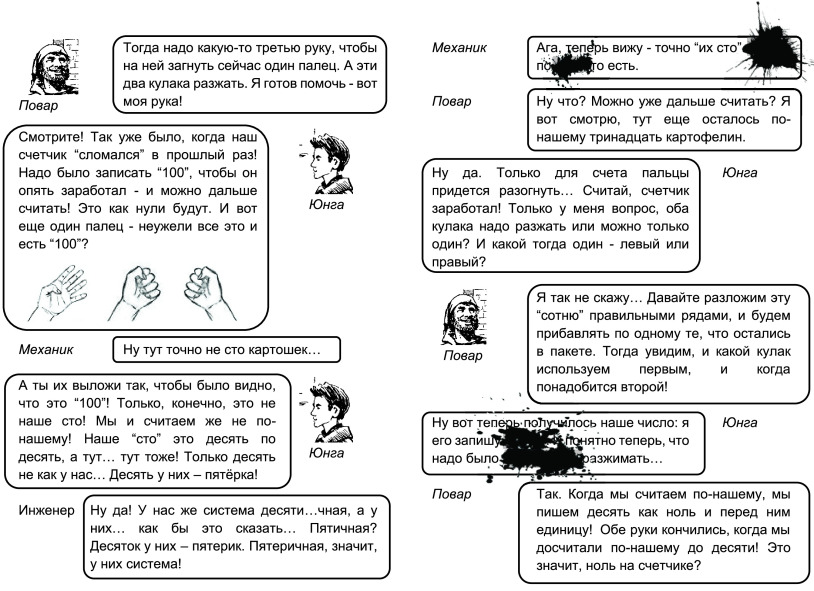
Students’ textbook — the travelers’ “Flight log” with ink stains

The students were invited to join a team of rescuers, who are following the trail of cosmic pioneers who have gone missing for a long time. The “Flight log” (*Figure 1*) has been passed along to the traveling rescue team with some fragments missing (coincidentally in the most important places) and describes the difficulties which the pioneers faced as they tried to communicate with alien-run refuel stations through their common decimal numbers. It contains dialogue fragments and some photos taken by the characters, written notes, and some of the destinations the pioneers were headed to.

**Figure 2.**
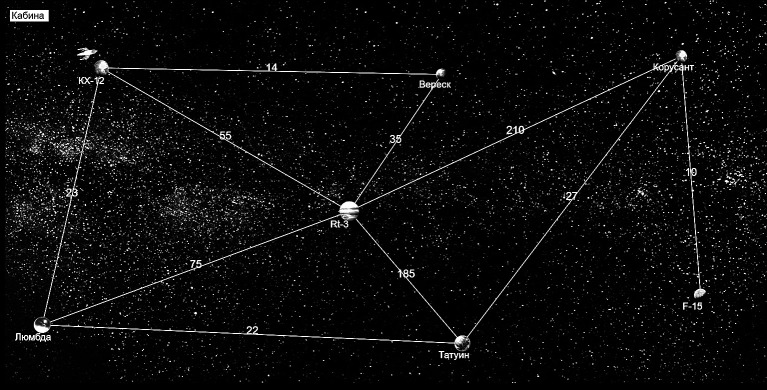
Map screen

*Note: the numbers on the map show the amount of fuel needed to travel from one destination to another (in Earth’s decimal counting). The spaceship (left top corner) marks player’s current position*

**Figure 3.**
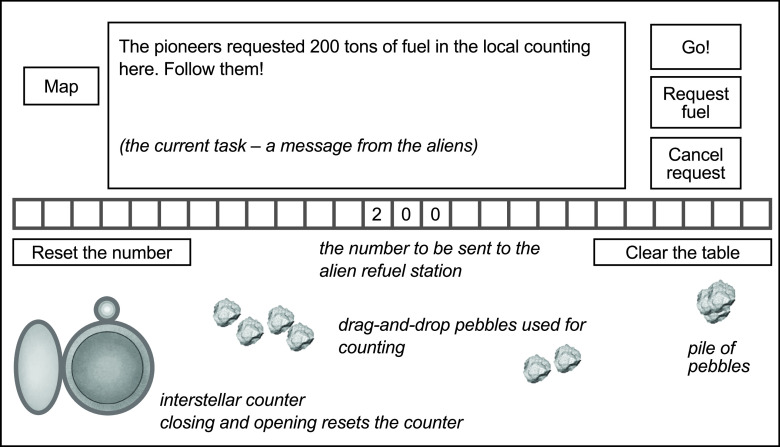
The Cabin view

Note: the screen was replicated from the real screen view in order to translate all text boxes from Russian

The computer simulation presents the sequence of “galaxies” which the pioneers have to travel through (the “Map” — see *Figure 2*) and the interface of the cabin screen with the slot for numbers, used to communicate with the refuel station in each “galaxy” on the way (the “Cabin view” — *Figure 3*). Students are to calculate the exact amounts of “cosmo-fuel” for different-base counting systems for each “cosmo-hop” which they make. If there is not enough, or too much fuel, the flight brings them nowhere — and they have to start anew.

We outline the students’ progress through the introductory “chapter” (the first of 12) of our educational module below:

The story starts as the pioneers request 23 tons of fuel at the cosmic refuel station for the destination they are heading to. However, the spaceship fails to reach that destination, and the pioneers find out that the aliens gave them only 11 tons of fuel. Did the aliens maliciously trick them? As the record of the dialogue between the characters in the flight journal shows, there was a “misunderstanding” due to aliens’ “exact understanding” of our numeral notation according to their “local” way of counting.

“We count just normally, like everyone does,” answers the alien when asked how they count. That is the key phrase, as it directs the pioneers, and hence the students who are “helping” them, toward analyzing the basis of their own counting system. Our experimental teaching showed that this phrase does not go unnoticed: students consider it a lie at first, as they obviously cannot get the needed amount of fuel with the number they dial. They will return to this phrase in the middle of the lesson, as they start to question what exactly there is in the aliens’ way of counting that is similar to our own. At the end of the chapter there were always students who would exclaim: “The aliens were right!”

The difficulties of communication with the refuel station only set the problem and the meaningful context, but do not yet pose the learning task itself. The task appears as students have to overcome the discrepancy between the special counting procedure with the tokens in a materialized modeling space, and the “natural” decimal counting system which uses familiar numerical notations. This new additional modeling space emerges as the characters (the pioneers) notice the “interstellar counter” in the cabin; this object looks like an old clock to Earth dwellers (*Figure 3*). However, there are no hands, and the clock face lights up and makes a sound for each touch. The aliens say that this device is used to count “worldwide.”

Text fragment #1.

Operator: The aliens say that it’s the interstellar counter. Each click on its face means “one.” They usually use some items for counting: they put one token and click once… Then the counter helps to write the number in the slots for the refuel station’s terminal.

Technician: We need to get twenty-three tons of fuel — what is there to count?

Skipper: No, we should still use the counter. Oh, there are even some pebbles here! Perhaps, they were left for us so we could use them for counting. Let us take twenty-three stones and…

Sea cadet: Well… We have done everything, as they said: put the stone — clicked the clock face, put the stone — clicked… But now it has ceased to count. The clock face glows and cannot be clicked again.

Technician: Perhaps, it broke down? Ask the aliens!

Operator: I showed them our counting table. They say that everything is okay: “It’s 100, that you have collected. Now you have to write 100 in the slots of the terminal and then you can count further.”

Captain: Do as they say!

Technician: We have written 100.

Engineer: It’s clear now! Of course, they gave us too little fuel. They already have 100, but we only have *** [stained with inks]. We were short for these many stones, I mean, tons of fuel: ***.

In the fragment above, the key plot element is set: counting “by the clock” cannot be continued after a certain amount, which is “100 in the local system.” It is here that counting is connected to the process of obtaining one “hundred.” Students have to perform counting just the way the characters (rescuers) did. Then they will know that the clock “broke” after sixteen counts, which means that “sixteen” in our decimal system is marked as “100” in the local counting system. Although the characters try to persuade the students to do the counting themselves (see the skipper’s and captain’s replies in fragment 1), our teaching experiments show that students at first do not follow the characters’ advice, but rather try to find ready “answers” in the text. They are obviously not ready for the type of digital support which demands actually operating the objects on the screen according to the text description of characters’ actions, which have no direct link with familiar “math activities.”

Even after repeating the first part of the operations needed (“put the pebble — click the clock (— put the pebble — click the clock” and so on until the clock “broke”), they could nd the 16 pebbles, but could not connect “our” sixteen and the alien “100.” They could grasp this connection only as they put 100 in the terminal slots (as the text tells them to) — and the “counter” stopped glowing and could be “clicked” again.

How should we count if we are to reach “100” on “our” sixteenth count? The aliens state that they count “like we do.” Students thus have to coordinate “our” common way of counting towards 100 with the given amount (16), which has to be re-counted to get alien’s “100.” It is here that reflection on the foundation of familiar counting becomes urgent: the “conceptual” content of place value in positional decimal counting is usually disguised with the “natural” appearance of decimal numeration, which has “10” after “9,” as there is no special “tenth” symbol. For other-base systems (less than decimal) we can easily suggest symbols for their “tens,” but for some reason they have to write “10” instead of 4 (in base-4), or instead of 7 (base-7 counting). In this respect our own decimal system thus seems the only “true” one, and others are regarded as weird derivatives.

“Disassembling” the new “hundred” to appropriate “tens” is introduced as the special action which will allow students to overcome their “obsessive” decimal counting attempts to calculate the needed fuel. This procedure will further be used for operating in the local numeral system, maintaining the consistency of counting, which is first revealed in the “hundreds” digit. Hence, the next step in the narrative is to “break” the 16 pebbles which comprised the aliens’ “hundred,” into bundles of “tens.” Various options, like 6 and 10, or 2 piles of 8, or 8 pairs, are rejected by the characters…

Text fragment #2.

Skipper: It is not evident here. You were collecting tokens — how did you say — ten times ten… we should try the same here.

Sea cadet: We don’t have enough pebbles for that! Even two times ten cannot be made with sixteen…

Skipper: They can. They also make “ten times ten,” so should we… But we have to reach their “100” sooner, than our “100.”

Sea cadet: Then the piles have to be smaller. Oh, I see… “this amount” by “the same amount”! As many piles as the number of pebbles in the pile. I gather now, how they count…

Technician: The aliens were right; they indeed count as we do. But their “ten” is not the same as our ten, it is *** in our words.

The students are left with 16 counting pebbles and the “stained” spot here. The essential words “this amount by the same amount” and “ten times ten” are in no way a direct instruction or a ready-made answer. The guidelines for students’ action — which would look like “arrange the 16 pebbles so that “ten times ten” makes 100” — are yet to be retrieved from these key words, which typically were ignored by all our participants at first. Therefore, the students test the idea of “system counting” in general, according to its orientational function. It is remarkable that the students were not able to solve this task (which perhaps looks simple for adults) until they took the counting material in their own hands (either actually or virtually) and started to put them in the piles. Students then had to tell each other and themselves “ten,” as they laid down four pebbles, and they were to repeat it up to “ten times ten” and lay down four piles of four pebbles each.

**Figure 4.**
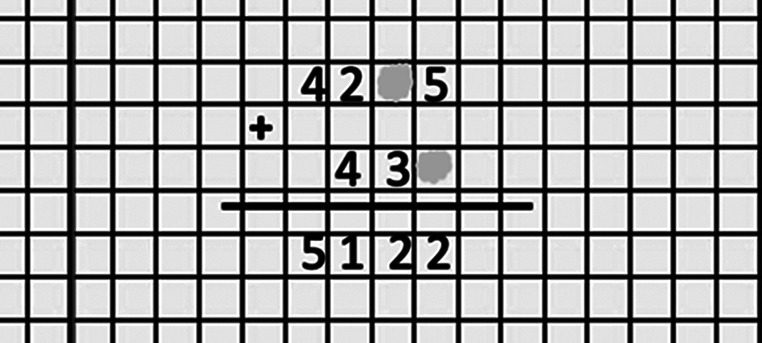
The scrap of paper with some missing numbers

Thus, here the orientation procedure is externalized as “piling up” the counting material. Students will appeal to this means during initial steps through the “Flight log” and the “training flights:” it will help them mediate their counting by the names and symbols used to operate multi-digit numbers. For example, the students will later on solve the arithmetic equations with “stained” numbers in other-base systems (*Figure 4*), which will demand the comprehension of the digit structure, and the appeal to different counting materials will serve as means for modeling (instead of ready-made illustrations of actions’ results typical of primary textbooks).

## Results

The built-in assessment of the reasonableness of students’ solutions was the analysis of students’ performance in the “training flights.” The logs of each students’ homework could be considered by the teachers and researchers, both qualitatively and quantitatively. In-depth analysis of the mistakes students made allowed us to distinguish between their attempts to apply the concept-mediated procedures learned in the class and random “guessing” strategies. The example of the latter is presented below (*Table 1*).

**Table 1:** Logs’ fragment: a student is conducting a training flight

Time	Action	*Our comment*
11:29:20	fly.. Oiro -> Tatuin .. success	*the previous successful flight*
11:31:28	fill up .. 18 .. fail	*fuel fillup failed, as there is no “8” in local base-3 system*
11:32:22	fill up .. 200 .. success	*“200” fuel in local counting was successfully obtained…*
11:32:23	fly.. Tatuin -> Argo .. fail	*… but the flight to Argo failed (it requires “200” in decimal counting)*
…	…	*12 more random attempts (failed)*
11:39:37	fill up .. 21 .. success	
11:39:43	fly трудный -> Apro .. fail	*student stops trying*

Task example 1 (the introductory chapter): “The aliens on the Tatuin say that the pioneers requested 200 tons of fuel in the local counting system and flew away. Where did they go?”

The students are supposed to open the “interstellar counter,” prepare the pebbles and count up till the counter stops to show local “100,” which will be our 9.

The map suggests several routes (in decimal counting): to Korusant (81), to Ymba (18), to Lumbda (109) and to Argo (200).

According to this part of log, we may assume that the student at first counted with the counter one “hundred” (nine pebbles) and then added one more “hundred” to get “200” (which is 18), but after that he tried to send the number “18” to the aliens, which they obviously could not accept. The student, perhaps, spent 1 minute reading the task again and then dialed “200” in the slots (which was indeed the alien number, which the pioneers also used). But as the student looked at the map, he was distracted by the Argo destination, which had “200” in decimal counting (a “pitfall” placed on purpose), chose it as the destination and failed. After that followed 13 random trials, but the task remained unsolved.

In this small fragment the student falls into the trap of “visuality” two times: first, he uses our “18” for the aliens, and then he searches for the alien “200” between decimal numbers on the map. Th rough the logs analysis, we can observe the progress of students who adopt the orientation procedure as their own means of acting within the digital simulation. Indeed, the log of this student on another day shows that he did this and other tasks in the series without false trials, and each problem took about 2 or 3 minutes for him to solve (to read and to perform the correct procedures).

The quantitative analysis to describe the progress of each student may use information about the time spent on the task, and the number of trials (successful and failed), which can be retrieved from the logs of students who successfully completed the tasks from the training flight with one or more attempts each.

The example also concerns tasks which demand choosing the destination which fits with the local number of cosmo-tons given. We can see from the log (see *Table 2*) that sorting through all variants may lead to success here. Yet, some students did not follow this path.

**Table 2:** The comparison of students’ “strategies”

	task #1		task #2		task #3		task #4	
Students	Time spent	Failed trials	Time spent	Failed trials	Time spent	Failed trials	Time spent	Failed trials
student F	0:57	0	9:31	1	4:47	0	3:29	0
student G	2:47	0	4:10	0	7:55	0	3:24	0
student H	2:14	1	6:38	1	6:04	1	1:33	1
student I	0:55	2	2:51	4	1:39	2	0:32	0
student J	1:43	0	0:36	0	1:55	4	1:41	1

*Note: “0” failed trials mean the destination was reached successfully on the first attempt.*

Task example 2: “The pioneers filled up 1000 tons of fuel in local counting here for their next cosmo-hop. Determine their destination and follow them!”

The “counter” stops on the 36th “click,” showing that the local “100” is the same as “our” 36. Thus, it is a base-6 system, and students have to take “ten” times “hundred” to get a “thousand”, which means 6 times 36 in our count.

Possible destinations: Luc (64), Marlo (1296), Tabu (216), Kani (360), and Rubicon (81).

As we see from the table, students I and J are spending very little time on each task and are doing a number of trials, which means that most likely they were guessing the answer, trying one destination after another. Sometimes they were lucky and succeeded with the first trial, and sometimes not. The other two students, F and G, apparently were those who performed the orientation procedures (arranging the pebbles to make “ten times ten,” recounting the necessary amount of fuel, etc.); they spent more time on each problem, but they made almost no failed trials. Student H was perhaps trying to solve the problem as required, but each time made some mistakes, which could be discussed and resolved during the next lesson.

An important indicator of the students’ progress throughout the module for educational designers and for the teachers was the decrease in the number of failed trials, especially the disappearance of “random guesses” (several failed trials performed within a small period of time). In our experimental teaching the number of “random” trials came down to an insignificant minimum. It is worth mentioning that the number of failed trials also decreased when the students were performing their tasks in groups rather than individually, and held a discussion (the teacher did not take part).

The tasks were specially modified throughout the development of the problem situation to keep the tension, in line with Galperin’s principles of active orientation scaffolding against stereotypical reactions and resorting to irrelevant “round-about” methods (Galperin, 1992, 2007; Engeness, 2021). Thus, the attempts sometimes failed due to the increasing difficulty of the tasks, and the time spent on each task could grow accordingly (student J, *Table 3*).

**Table 3:** Systemized logs’ information: solving a series of tasks of increased difficulty level

	task #1		task #2		task #3		task #4	
Students	Time spent	Failed trials	Time spent	Failed trials	Time spent	Failed trials	Time spent	Failed trials
student L	1:20	0	4:20	0	1:53	1	2:37	1
student M	1:21	0	9:08	0	0:53	0	3:22	1
student N	1:34	0	16:34	5	2:32	0	18:40	6

*Note: “0” failed trials means the destination was reached successfully on the first attempt.*

Task example 3 (the last chapter). For the next cosmo-hop the pioneers tried to guess the local system and wrote down the number of cosmo-tons in the base-2 numeration: “111001”. However, the aliens from the current refuel station said: “Are you sure? It is enormously far from here. Bear in mind that your “five” fingers, as you call them, is “12” fingers in our counting. Request the appropriate amount of fuel and proceed further.”

Possible destinations: Crocus (11), Jot (111), Het (5), Zews (57), Dub (49).

The results of the teaching series proved the potential of the content and the computer support which we have developed. The educational module scaffolded students’ progress in a way which made it possible to proceed with each following lesson, as an extension of the previous one. Moreover, the students themselves could set the new task, as they were immersed in the content and could see the further development and consequences of their current problem. The story of the cosmic pioneers gradually faded into the background and the students got more involved in the objective relations within the numeral systems. Since the contradictions between “how it seems” and “how it should be done according to the concept” were themselves maintaining the tension, the characters’ statements were only making these contradictions more vivid. There was no need for “extra external motivation” to keep students busy with problem-solving. The substantial progress of our students within the regular mathematics curriculum was confirmed by an expert assessment and their performance on common written math tests on corresponding topics.

## Discussion

The digital environment which we have designed for the “Interstellar wanderers” module, provided essential support for setting up students’ substantial reflection on the structure and functionality of positional counting as what Davydov called the specific learning task (Davydov, 2008). The appeal to orientation procedures within a special modelling space (the counting of tokens, which problematized the use of current digits’ measures) was supported constantly through the educational text. The narration of the “Flight log” provided hints and clues on the required orientation procedures according to their fundamental functions, and set them as the means of communication between the characters about the problems which arose along the way. Trivial but false ideas were uttered by the “crew members” to prompt the students: they represented the tempting “roundabout” easy ways of handling the matter, which are to be rejected on the basis of “concept-mediated” actions. Each cosmo-hop not only served to check the answer, but allowed further development of the learning situation.

Control over the correctness of the answer was moved to the special modelling space: there students checked the reasonableness of the way they presented this or that number in decimal or other-base counting. Thus, students refused to check some answers unless there was substantial modelling behind them. It was the method of modelling, not the answer itself, which had to be verified. The “abrupt” introduction of an alien “hundred” as the digit which concealed and at the same time allowed the general structure of the multi-digit notation to unfold, showed vast potential to immerse students into the contradictions between the “conceptual” and “familiar” within the positional counting. Students thus learned to evade popular mistakes in everyday common calculations in the decimal system.

The simulation of the “intergalactic” flights as presented, created a special environment wherein the students were comparatively free in their actions, had clear goals, and were confronted by real challenges which did not depend on the teacher’s will. The teacher was not there to judge the correctness of the students’ answers; on the contrary, when the students asked for the teacher’s comment and opinion (“Am I doing it right?”), he had them return to the content of the situation instead: “Look, you haven’t reached this galaxy.” He might even prompt wrong decisions and confuse the students, so that after their joint failure, they would have to teach the teacher the conceptual way to handle the matter. The productiveness of the students’ discussions and their progress, which happened as it was intended to, indicate the relevance of the designed content for supporting the students’ model actions, and justifies the chosen approach to the digitalization of school lessons.

## Conclusion

The prospects of providing common educational content by digital means seem doubtful; making traditional visual aids more and more vivid and eye-catching has not yet brought any “breakthrough” in students’ comprehension of place value in particular. On the contrary, the approach to computer support design based on the general principles of Activity Theory in education has once again proved to be feasible and productive, although it does not produce the same abundance of software samples.

The basic principles of digital environment design, which have already been tested in our previous studies (Rubtsov, & Ulanovskaya, 2021; Vysotskaya et al., 2017; Vysotskaya, Lobanova, & Yanishevskaya, 2022), are: support for students’ purposeful transformations performed with objects, and the organization of special additional modelling space on the screen. However, these principles can be applied only after the concept’s origin has been reconstructed, and a feasible hypothesis on the content of students’ appropriate orientation procedures has been formulated. The digitalization provides new opportunities for conducting a lesson, but bears the substantial risk of strengthening students’ mindless attempts to knock the answer readymade from the computer, without delving deep into the matter (“gaming the system,” according to Richey et al., 2021). In this regard, the analysis of the concepts’ content and origin is even more urgent for the design of educational modules with computer support.

Students’ engagement in the content of the school disciplines itself through specially designed digital environments remains an urgent issue (Belolutskaya, Vachkova, & Patarakin, 2023). We assume that our approach may extend teachers’ opportunities for using digital environments (“gaming,” in particular) for educational purposes (Lampropoulos, 2023; Papadakis, Zourmpakis, & Kalogiannakis, 2023; Ruthven, Hennessy, & Brindley, 2004; Zourmpakis, Papadakis, & Kalogiannakis, 2022). Therefore, further investigation should describe in detail how this approach promotes students’ progress in conceptual reasoning.

## Limitations

In the pilot project, we focused most on the correction of learning materials and update of the software’s functionality. Thus, serious testing of the learning module on a bigger sample, and its refinement for use by a regular teacher is the goal of our future research.

## Footnotes

1 Some examples of internet sources:
https://www.topmarks.co.uk/maths-games/7-11-years/place-value

https://ictgames.com/mobilePage/getToKnow/index.html

https://phet.colorado.edu/en/simulations/number-play
